# The Prognostic Value of TP53 Alteration in Patients with Head and Neck Squamous Cell Carcinoma Receiving Immunotherapy

**DOI:** 10.1093/oncolo/oyac087

**Published:** 2022-05-10

**Authors:** Chao Jiang, Xuanchen Zhou, Jie Han, Zhiyong Yue, Butuo Li

**Affiliations:** Department of Otorhinolaryngology Head and Neck Surgery, Shandong Provincial Hospital Affiliated to Shandong First Medical University; Shandong Provincial Hospital, Cheeloo College of Medicine, Shandong University, Jinan, Shandong 250021, People’s Republic of China; Department of Otorhinolaryngology Head and Neck Surgery, Shandong Provincial Hospital Affiliated to Shandong First Medical University; Shandong Provincial Hospital, Cheeloo College of Medicine, Shandong University, Jinan, Shandong 250021, People’s Republic of China; Department of Otorhinolaryngology Head and Neck Surgery, Shandong Provincial Hospital Affiliated to Shandong First Medical University; Shandong Provincial Hospital, Cheeloo College of Medicine, Shandong University, Jinan, Shandong 250021, People’s Republic of China; Department of Otorhinolaryngology Head and Neck Surgery, Shandong Provincial Hospital Affiliated to Shandong First Medical University; Shandong Provincial Hospital, Cheeloo College of Medicine, Shandong University, Jinan, Shandong 250021, People’s Republic of China; Department of Radiation Oncology, Shandong Cancer Hospital and Institute, Shandong First Medical University and Shandong Academy of Medical Science, Jinan 250117, Shandong Province, People’s Republic of China

## Abstract

The article by Wilson et al., published in the February 2021 issue, reported the prognostic value of circulating tumor DNA (ctDNA) sequencing in head and neck squamous cell carcinoma (HNSCC), noting TP53 as the most altered and concordant gene in ctDNA and tDNA. This Letter to the Editor further considers the role of TP53 alteration in the prognosis prediction of immunotherapy in HNSCC.

In *The Oncologist*, Wilson et al reported the prognostic value of circulating tumor DNA (ctDNA) sequencing in head and neck squamous cell carcinoma (HNSCC) for the first time.^1^ Of interest, TP53 was the most altered gene (73.3%) and the most concordant gene in ctDNA and tDNA. The authors also found the significant association between TP53 alteration and inferior survival of HNSCC patients, which may be of significant utility in precision oncology treatment strategies including target agents and immunotherapy.

Thus, we further focused on the predictive value of the TP53 alteration in immunotherapy.

Pembrolizumab and nivolumab have been approved for the treatment of HNSCC patients based on the CheckMate141, KEYNOTE-012, and KEYNOTE-048 trials.^2^ However, the clinical benefit to immunotherapy is limited to less than 20%.^3^ Thus, the discovery of predictive biomarkers is critical to optimize treatment strategies and improve survival of HNSCC patients. TP53 alteration has been found to be associated with activated T-effector and interferon-γ signature and expression of immune checkpoints including PD-L1^4^ and has been represented to be the potential biomarker for lung adenocarcinoma patients receiving immunotherapy.^4^ We believe the prognostic value of TP53 alteration for HNSCC patients receiving immunotherapy is worth further discussion.

Here, we evaluated the relationship between TP53 alteration and response to immunotherapy among patients in tumor mutational burden (TMB) and immunotherapy database, which included clinical and genomic data from next-generation sequencing of 1662 patients with advanced cancer receiving immunotherapy. The prevalence of TP53 alteration is 44.4% (738/1661) in the entire cohort and is the most common in HNSCC cohort (58/128, 45.3%). We further evaluated the prognostic value of TP53 alteration for immunotherapy. The results indicate altered TP53 is associated with inferior overall survival (OS) in the entire cohort (median OS, 14 vs 22 months, *P* < .001; Figure 1A). Similar result is observed in HNSCC cohort, median OS is 8 months among patients with TP53 alteration compared with 14 months in unaltered group (*P* = .0318; Figure 1B).

**Figure 1.**
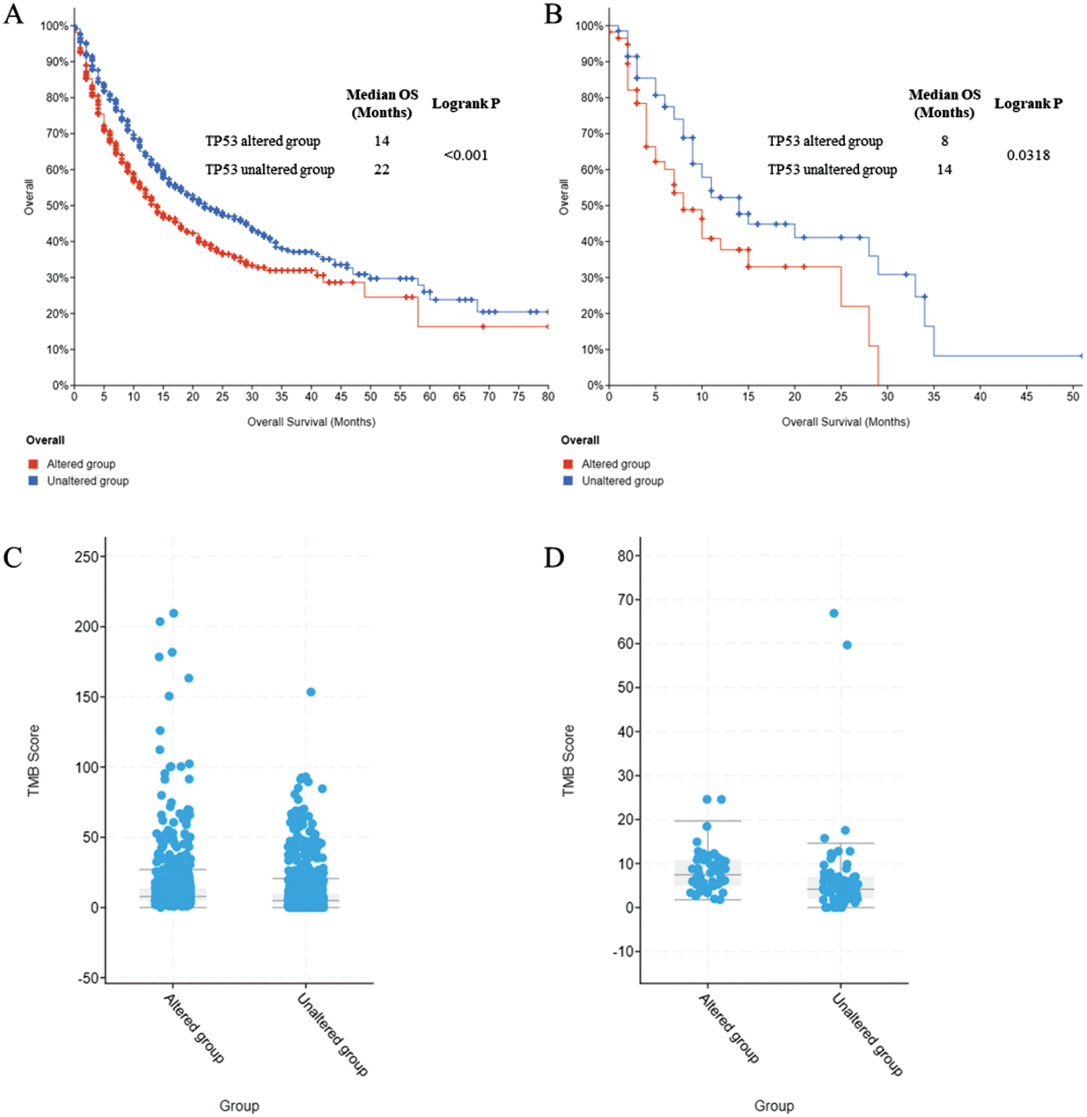
The survival curves of TP53 alteration for immunotherapy and association between TP53 alteration and TMB score. **(A):** Difference of KM survival curves of overall survival (OS) between TP53 altered and unaltered group in the entire cohort. **(B):** Difference of KM survival curves of overall survival (OS) between TP53 altered and unaltered group in the HNSCC cohort. **(C):** Difference of TMB score between TP53 altered and unaltered group in the entire cohort. **(D):** Difference of TMB score between TP53 altered and unaltered group in the HNSCC cohort.

Based on the valuable role of TMB for the prognostic prediction of immunotherapy, we further performed the Kruskal-Wallis test to assess the association between TMB score and TP53 alteration, and significant positive correlation was observed in the entire cohort (median score, 7.87 vs 4.92, *P* < .001; Figure 1C). Besides, TMB score was also significantly higher in patients with HNSCC harboring TP53 alteration receiving immunotherapy (median score, 7.45 vs 4.16, *P* < .001; Figure 1D).

Our results further indicate the prognostic value of TP53 alteration for immunotherapy. Another interesting finding is the positive correlation between TP53 alteration and high TMB score. Wilson et al also found the concordance of TP53 alteration between tDNA and ctDNA sequencing in HNSCC. Thus, this study also provides new insight that ctDNA alterations in TP53 might be served as convenient biomarkers for immunotherapy in patients with HNSCC, and further validations is needed for clinical practice.
